# Hexa-μ_2_-acetato-1:2κ^4^ *O*:*O*′;1:2κ^2^ *O*:*O*;2:3κ^4^ *O*:*O*′;2:3κ^2^ *O*:*O*-bis­(2-amino-7-chloro-5-methyl-1,8-naphthyridine)-1κ*N* ^1^,3κ*N* ^1^-trizinc(II)

**DOI:** 10.1107/S1600536808024549

**Published:** 2008-08-06

**Authors:** Xin-Sheng Li, Juan Mo, Li Yuan, Jian-Hua Liu, Su-Mei Zhang

**Affiliations:** a College of Animal Husbandry and Veterinary Studies, Henan Agricultural University, Zhengzhou, Henan Province 450002, People’s Republic of China

## Abstract

The title complex, [Zn_3_(C_2_H_3_O_2_)_6_(C_9_H_8_ClN_3_)_2_], contains three Zn^II^ atoms bridged by six acetate ligands. The central Zn^II^ ion, located on an inversion centre, is surrounded by six O atoms from acetate ligands in a distorted octa­hedral geometry [Zn—O = 1.9588 (12)–2.1237 (12) Å]. The terminal Zn^II^ ions are coordinated by one N atom of 2-amino-7-chloro-5-methyl-1,8-naphthyridine and three O atoms of three acetate ligands in a distorted tetra­hedral geometry. The separation between the central and terminal Zn^II^ ions is 3.245 (3) Å.

## Related literature

For related literature, see: Baker & Norman (2004 ▶); Lis *et al.* (2005 ▶); Stadie *et al.* (2007 ▶).
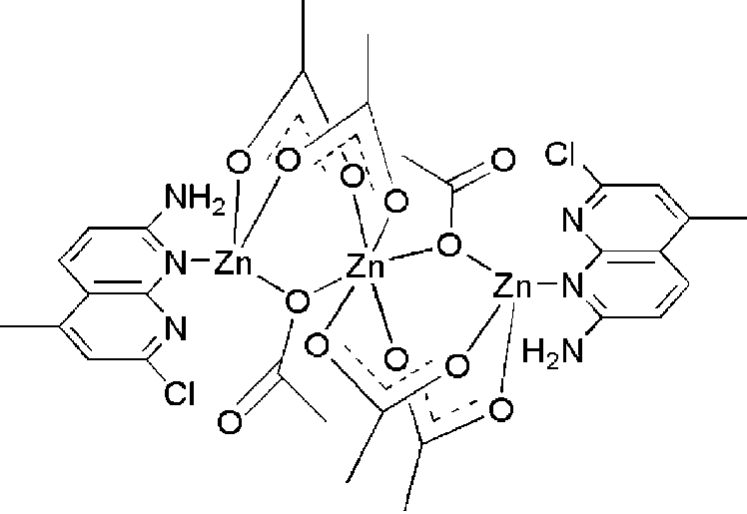


## Experimental

### 

#### Crystal data

[Zn_3_(C_2_H_3_O_2_)_6_(C_9_H_8_ClN_3_)_2_]
*M* *_r_* = 937.64
Triclinic, 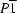

*a* = 9.1978 (12) Å
*b* = 9.2108 (13) Å
*c* = 12.0457 (16) Å
α = 93.602 (3)°
β = 91.685 (2)°
γ = 118.247 (2)°
*V* = 895.2 (2) Å^3^
*Z* = 1
Mo *K*α radiation
μ = 2.21 mm^−1^
*T* = 113 (2) K
0.12 × 0.08 × 0.02 mm

#### Data collection

Bruker SMART CCD area-detector diffractometer
Absorption correction: multi-scan (*SADABS*; Sheldrick, 1996 ▶) *T*_min_ = 0.782, *T*_max_ = 0.956
11008 measured reflections
4221 independent reflections
3495 reflections with *I* > 2σ(*I*)
*R*_int_ = 0.030

#### Refinement

*R*[*F* ^2^ > 2σ(*F* ^2^)] = 0.024
*wR*(*F* ^2^) = 0.065
*S* = 1.01
4221 reflections
253 parameters
H atoms treated by a mixture of independent and constrained refinement
Δρ_max_ = 0.36 e Å^−3^
Δρ_min_ = −0.65 e Å^−3^

### 

Data collection: *SMART* (Bruker, 1997 ▶); cell refinement: *SAINT* (Bruker, 1997 ▶); data reduction: *SAINT*; program(s) used to solve structure: *SHELXS97* (Sheldrick, 2008 ▶); program(s) used to refine structure: *SHELXL97* (Sheldrick, 2008 ▶); molecular graphics: *XP* in *SHELXTL* (Sheldrick, 2008 ▶); software used to prepare material for publication: *XP* in *SHELXTL*.

## Supplementary Material

Crystal structure: contains datablocks global, I. DOI: 10.1107/S1600536808024549/hg2413sup1.cif

Structure factors: contains datablocks I. DOI: 10.1107/S1600536808024549/hg2413Isup2.hkl

Additional supplementary materials:  crystallographic information; 3D view; checkCIF report

## Figures and Tables

**Table:** 

Zn1—O3	2.0724 (12)
Zn1—O5	2.0910 (12)
Zn1—O1	2.1237 (12)
Zn2—O3	1.9588 (12)
Zn2—O2	1.9748 (12)
Zn2—N2	2.0379 (14)

**Table:** 

O3—Zn1—O5^i^	91.21 (5)
O3—Zn1—O5	88.79 (5)
O3—Zn1—O1^i^	89.96 (5)
O5—Zn1—O1^i^	93.21 (5)
O3—Zn1—O1	90.04 (5)
O5—Zn1—O1	86.79 (5)

Symmetry code: (i) 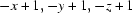.

**Table 2:** Hydrogen-bond geometry (Å, °)

*D*—H⋯*A*	*D*—H	H⋯*A*	*D*⋯*A*	*D*—H⋯*A*
N3—H3*B*⋯O2	0.85 (2)	2.15 (2)	2.921 (2)	150.0 (17)
N3—H3*A*⋯O5^ii^	0.87 (2)	2.09 (2)	2.958 (2)	171.0 (19)

Symmetry code: (ii) 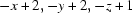.

**Fig. 1.**
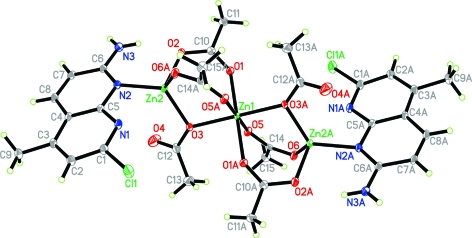
Molecular structure of the title compound showing the atom-numbering scheme and displacement ellipsoids drawn at the 40% probability level. (Symmetry code: 1 - x, 1 - y, 1 - z).

## supplementary crystallographic information

### Comment

Acetic acid is versatile ligand which can function in monodentate or bidentate modes in metal complexes (Baker & Norman, 2004; Lis *et al.*, 2005; Stadie *et al.*, 2007). Here, we report the crystal structure of the title compound, (I), in which three Zn^II^ ions are bridged by three acetic acid ligands.

The middle Zn atom in (I) (Fig. 1) has a distorted octahedral coordination geometry involving six O atoms of six acetic acid ligands. The Zn—O bonds lengths are between 1.9588 (12) and 2.1237 (12)Å (Table 1). The End Zn^II^ ion is coordinated by one N atom of 2-amino-4-methyl-7-chloro-1,8-naphthyridine and three O atoms of three acetic acid ligands, and has a distorted tetrahedron coordination geometry. The distance of two neighboring Zn^II^ ions separated by two O atoms of each two acetate bridging ligands and one O atoms of one acetate bridging ligand is 3.245 (3) Å.

### Experimental

A 10 ml dichloromethane solution of 2-amino-5-methyl-7-chloro-1,8-naphthyridine (0.039 g, 0.2 mmol) was added to a 20 mL dichloromethane solution of Zn(CH_3_COO)_2_ (0.055 g, 0.3 mmol) under an N_2_ atmosphere. The mixture was stirred for 10 h. Colorless crystals suitable for X-ray diffraction were formed by vapour diffusion of diethyl ethyl ether into dichloromethane solution.

### Refinement

All hydrogen atoms were generated geometrically (C—H bond lengths of methyl group fixed at 0.98 Å, C—H bond lengths of naphthyridine fixed at 0.95 Å), assigned appropriated isotropic thermal parameters, *U*_iso_(H) = 1.2*U*_eq_(C).

### Crystal data

**Table:**

[Zn_3_(C_2_H_3_O_2_)_6_(C_9_H_8_ClN_3_)_2_]	*Z* = 1
*M**_r_* = 937.64	*F*_000_ = 476
Triclinic, *P*1	*D*_x_ = 1.739 Mg m^−^^3^
Hall symbol: -P 1	Mo *K*α radiation λ = 0.71070 Å
*a* = 9.1978 (12) Å	Cell parameters from 2625 reflections
*b* = 9.2108 (13) Å	θ = 2.5–25.0º
*c* = 12.0457 (16) Å	µ = 2.21 mm^−^^1^
α = 93.602 (3)º	*T* = 113 (2) K
β = 91.685 (2)º	Prism, colorless
γ = 118.247 (2)º	0.12 × 0.08 × 0.02 mm
*V* = 895.2 (2) Å^3^	

### Data collection

**Table:**

Bruker SMART CCD area-detector diffractometer	4221 independent reflections
Radiation source: fine-focus sealed tube	3495 reflections with *I* > 2σ(*I*)
Monochromator: graphite	*R*_int_ = 0.030
*T* = 113(2) K	θ_max_ = 27.9º
φ and ω scans	θ_min_ = 2.5º
Absorption correction: multi-scan(SADABS; Sheldrick, 1996)	*h* = −12→12
*T*_min_ = 0.782, *T*_max_ = 0.956	*k* = −12→12
11008 measured reflections	*l* = −15→15

### Refinement

**Table:**

Refinement on *F*^2^	Secondary atom site location: difference Fourier map
Least-squares matrix: full	Hydrogen site location: inferred from neighbouring sites
*R*[*F*^2^ > 2σ(*F*^2^)] = 0.024	H atoms treated by a mixture of independent and constrained refinement
*wR*(*F*^2^) = 0.065	*w* = 1/[σ^2^(*F*_o_^2^) + (0.0354*P*)^2^] where *P* = (*F*_o_^2^ + 2*F*_c_^2^)/3
*S* = 1.01	(Δ/σ)_max_ = 0.001
4221 reflections	Δρ_max_ = 0.36 e Å^−^^3^
253 parameters	Δρ_min_ = −0.65 e Å^−^^3^
Primary atom site location: structure-invariant direct methods	Extinction correction: none

### Special details

**Table:**

Geometry. All e.s.d.'s (except the e.s.d. in the dihedral angle between two l.s. planes) are estimated using the full covariance matrix. The cell e.s.d.'s are taken into account individually in the estimation of e.s.d.'s in distances, angles and torsion angles; correlations between e.s.d.'s in cell parameters are only used when they are defined by crystal symmetry. An approximate (isotropic) treatment of cell e.s.d.'s is used for estimating e.s.d.'s involving l.s. planes.
Refinement. Refinement of *F*^2^ against ALL reflections. The weighted *R*-factor *wR* and goodness of fit *S* are based on *F*^2^, conventional *R*-factors *R* are based on *F*, with *F* set to zero for negative *F*^2^. The threshold expression of *F*^2^ > σ(*F*^2^) is used only for calculating *R*-factors(gt) *etc*. and is not relevant to the choice of reflections for refinement. *R*-factors based on *F*^2^ are statistically about twice as large as those based on *F*, and *R*- factors based on ALL data will be even larger.

### Fractional atomic coordinates and isotropic or equivalent isotropic displacement parameters (Å^2^)

**Table:**

	*x*	*y*	*z*	*U*_iso_*/*U*_eq_	
Zn1	0.5000	0.5000	0.5000	0.01215 (8)	
Zn2	0.81766 (2)	0.76297 (2)	0.660723 (16)	0.01398 (7)	
Cl1	0.47109 (6)	0.69320 (6)	0.93855 (4)	0.03122 (13)	
N1	0.75010 (17)	0.85718 (17)	0.85071 (12)	0.0158 (3)	
N2	0.97997 (17)	0.97283 (17)	0.75522 (12)	0.0136 (3)	
N3	1.20715 (19)	1.0819 (2)	0.64915 (13)	0.0181 (3)	
H3A	1.299 (3)	1.168 (3)	0.6360 (17)	0.024 (6)*	
H3B	1.164 (2)	0.998 (2)	0.6007 (17)	0.018 (5)*	
C1	0.6629 (2)	0.8670 (2)	0.93115 (15)	0.0179 (4)	
C2	0.7103 (2)	1.0020 (2)	1.01047 (15)	0.0182 (4)	
H2	0.6394	0.9999	1.0669	0.022*	
C3	0.8637 (2)	1.1386 (2)	1.00414 (14)	0.0163 (4)	
C4	0.9626 (2)	1.1348 (2)	0.91812 (14)	0.0143 (3)	
C5	0.8998 (2)	0.9917 (2)	0.84338 (14)	0.0138 (3)	
C6	1.1288 (2)	1.0979 (2)	0.73619 (14)	0.0140 (4)	
C7	1.2028 (2)	1.2477 (2)	0.80965 (15)	0.0174 (4)	
H7	1.3084	1.3350	0.7962	0.021*	
C8	1.1228 (2)	1.2645 (2)	0.89753 (15)	0.0170 (4)	
H8	1.1731	1.3632	0.9462	0.020*	
C9	0.9208 (2)	1.2883 (2)	1.08722 (16)	0.0222 (4)	
H9A	0.9206	1.3797	1.0499	0.033*	
H9B	1.0331	1.3213	1.1178	0.033*	
H9C	0.8458	1.2608	1.1478	0.033*	
C10	0.8902 (2)	0.6534 (2)	0.45903 (14)	0.0152 (4)	
C11	1.0029 (2)	0.6306 (2)	0.38019 (16)	0.0206 (4)	
H11A	1.0654	0.5849	0.4187	0.031*	
H11B	1.0799	0.7376	0.3545	0.031*	
H11C	0.9369	0.5543	0.3160	0.031*	
C12	0.5768 (2)	0.8498 (2)	0.60578 (15)	0.0195 (4)	
C13	0.4045 (2)	0.8233 (3)	0.59278 (19)	0.0314 (5)	
H13A	0.3986	0.9177	0.6303	0.047*	
H13B	0.3298	0.7220	0.6261	0.047*	
H13C	0.3714	0.8128	0.5134	0.047*	
C14	0.3533 (2)	0.5867 (2)	0.29542 (14)	0.0132 (3)	
C15	0.3642 (2)	0.7221 (2)	0.22734 (15)	0.0200 (4)	
H15A	0.3524	0.8054	0.2753	0.030*	
H15B	0.2755	0.6755	0.1677	0.030*	
H15C	0.4716	0.7738	0.1946	0.030*	
O1	0.73730 (14)	0.56434 (14)	0.44255 (10)	0.0174 (3)	
O2	0.95980 (15)	0.76262 (15)	0.54110 (10)	0.0191 (3)	
O3	0.59970 (14)	0.72059 (14)	0.60042 (10)	0.0170 (3)	
O4	0.69633 (18)	0.98977 (17)	0.62159 (15)	0.0385 (4)	
O5	0.46887 (14)	0.62359 (14)	0.36908 (10)	0.0165 (3)	
O6	0.22937 (14)	0.44604 (14)	0.27489 (10)	0.0178 (3)	

### Atomic displacement parameters (Å^2^)

**Table:**

	*U*^11^	*U*^22^	*U*^33^	*U*^12^	*U*^13^	*U*^23^
Zn1	0.01022 (14)	0.01115 (14)	0.01260 (15)	0.00360 (11)	−0.00109 (10)	−0.00257 (11)
Zn2	0.01081 (11)	0.01109 (11)	0.01601 (12)	0.00248 (8)	−0.00174 (8)	−0.00236 (8)
Cl1	0.0224 (2)	0.0204 (2)	0.0333 (3)	−0.0039 (2)	0.0130 (2)	−0.0047 (2)
N1	0.0152 (7)	0.0122 (7)	0.0145 (8)	0.0023 (6)	−0.0001 (6)	−0.0009 (6)
N2	0.0133 (7)	0.0104 (7)	0.0141 (7)	0.0034 (6)	0.0006 (6)	0.0003 (6)
N3	0.0130 (8)	0.0141 (8)	0.0220 (9)	0.0021 (7)	0.0041 (6)	0.0008 (7)
C1	0.0159 (9)	0.0140 (9)	0.0173 (9)	0.0017 (7)	0.0027 (7)	0.0013 (7)
C2	0.0204 (9)	0.0165 (9)	0.0155 (9)	0.0070 (8)	0.0031 (7)	0.0012 (7)
C3	0.0204 (9)	0.0144 (9)	0.0145 (9)	0.0091 (8)	−0.0036 (7)	−0.0009 (7)
C4	0.0143 (8)	0.0112 (8)	0.0153 (9)	0.0049 (7)	−0.0038 (7)	−0.0006 (6)
C5	0.0134 (8)	0.0128 (8)	0.0135 (9)	0.0051 (7)	−0.0013 (7)	0.0009 (7)
C6	0.0128 (8)	0.0110 (8)	0.0170 (9)	0.0049 (7)	−0.0020 (7)	0.0011 (7)
C7	0.0118 (8)	0.0103 (8)	0.0248 (10)	0.0012 (7)	−0.0015 (7)	0.0002 (7)
C8	0.0159 (9)	0.0097 (8)	0.0214 (9)	0.0036 (7)	−0.0039 (7)	−0.0024 (7)
C9	0.0241 (10)	0.0176 (9)	0.0215 (10)	0.0081 (8)	−0.0021 (8)	−0.0053 (8)
C10	0.0165 (8)	0.0127 (8)	0.0164 (9)	0.0065 (7)	0.0019 (7)	0.0038 (7)
C11	0.0164 (9)	0.0215 (9)	0.0231 (10)	0.0083 (8)	0.0052 (7)	0.0011 (8)
C12	0.0227 (9)	0.0160 (9)	0.0190 (10)	0.0091 (8)	−0.0009 (7)	−0.0018 (7)
C13	0.0235 (10)	0.0231 (11)	0.0490 (14)	0.0136 (9)	−0.0034 (10)	−0.0060 (9)
C14	0.0136 (8)	0.0141 (8)	0.0128 (8)	0.0073 (7)	0.0042 (6)	−0.0010 (7)
C15	0.0206 (9)	0.0181 (9)	0.0201 (10)	0.0078 (8)	0.0021 (7)	0.0058 (7)
O1	0.0117 (6)	0.0176 (6)	0.0191 (7)	0.0044 (5)	0.0018 (5)	−0.0026 (5)
O2	0.0139 (6)	0.0172 (6)	0.0195 (7)	0.0027 (5)	0.0008 (5)	−0.0041 (5)
O3	0.0150 (6)	0.0134 (6)	0.0223 (7)	0.0079 (5)	−0.0053 (5)	−0.0076 (5)
O4	0.0261 (8)	0.0178 (7)	0.0673 (11)	0.0077 (6)	−0.0081 (7)	0.0025 (7)
O5	0.0133 (6)	0.0132 (6)	0.0179 (7)	0.0022 (5)	−0.0016 (5)	0.0020 (5)
O6	0.0151 (6)	0.0125 (6)	0.0214 (7)	0.0037 (5)	−0.0038 (5)	−0.0014 (5)

### Geometric parameters (Å, °)

**Table:**

Zn1—O3	2.0724 (12)	C7—C8	1.349 (2)
Zn1—O3^i^	2.0724 (12)	C7—H7	0.9500
Zn1—O5^i^	2.0910 (12)	C8—H8	0.9500
Zn1—O5	2.0910 (12)	C9—H9A	0.9800
Zn1—O1^i^	2.1237 (12)	C9—H9B	0.9800
Zn1—O1	2.1237 (12)	C9—H9C	0.9800
Zn2—O3	1.9588 (12)	C10—O1	1.249 (2)
Zn2—O2	1.9748 (12)	C10—O2	1.275 (2)
Zn2—O6^i^	1.9783 (12)	C10—C11	1.504 (2)
Zn2—N2	2.0379 (14)	C11—H11A	0.9800
Cl1—C1	1.7428 (18)	C11—H11B	0.9800
N1—C1	1.302 (2)	C11—H11C	0.9800
N1—C5	1.358 (2)	C12—O4	1.234 (2)
N2—C6	1.346 (2)	C12—O3	1.301 (2)
N2—C5	1.359 (2)	C12—C13	1.487 (2)
N3—C6	1.329 (2)	C13—H13A	0.9800
N3—H3A	0.87 (2)	C13—H13B	0.9800
N3—H3B	0.85 (2)	C13—H13C	0.9800
C1—C2	1.401 (2)	C14—O6	1.259 (2)
C2—C3	1.385 (2)	C14—O5	1.264 (2)
C2—H2	0.9500	C14—C15	1.502 (2)
C3—C4	1.408 (2)	C15—H15A	0.9800
C3—C9	1.512 (2)	C15—H15B	0.9800
C4—C5	1.407 (2)	C15—H15C	0.9800
C4—C8	1.433 (2)	O6—Zn2^i^	1.9783 (12)
C6—C7	1.439 (2)		
			
O3—Zn1—O3^i^	180.0	C8—C7—C6	120.23 (16)
O3—Zn1—O5^i^	91.21 (5)	C8—C7—H7	119.9
O3^i^—Zn1—O5^i^	88.79 (5)	C6—C7—H7	119.9
O3—Zn1—O5	88.79 (5)	C7—C8—C4	120.55 (16)
O3^i^—Zn1—O5	91.21 (5)	C7—C8—H8	119.7
O5^i^—Zn1—O5	180.000 (1)	C4—C8—H8	119.7
O3—Zn1—O1^i^	89.96 (5)	C3—C9—H9A	109.5
O3^i^—Zn1—O1^i^	90.04 (5)	C3—C9—H9B	109.5
O5^i^—Zn1—O1^i^	86.79 (5)	H9A—C9—H9B	109.5
O5—Zn1—O1^i^	93.21 (5)	C3—C9—H9C	109.5
O3—Zn1—O1	90.04 (5)	H9A—C9—H9C	109.5
O3^i^—Zn1—O1	89.96 (5)	H9B—C9—H9C	109.5
O5^i^—Zn1—O1	93.21 (5)	O1—C10—O2	124.22 (16)
O5—Zn1—O1	86.79 (5)	O1—C10—C11	119.27 (15)
O1^i^—Zn1—O1	180.0	O2—C10—C11	116.48 (15)
O3—Zn2—O2	111.65 (5)	C10—C11—H11A	109.5
O3—Zn2—O6^i^	103.18 (5)	C10—C11—H11B	109.5
O2—Zn2—O6^i^	100.45 (5)	H11A—C11—H11B	109.5
O3—Zn2—N2	124.29 (5)	C10—C11—H11C	109.5
O2—Zn2—N2	99.92 (5)	H11A—C11—H11C	109.5
O6^i^—Zn2—N2	115.01 (6)	H11B—C11—H11C	109.5
C1—N1—C5	116.26 (15)	O4—C12—O3	120.09 (17)
C6—N2—C5	118.94 (14)	O4—C12—C13	121.64 (17)
C6—N2—Zn2	132.49 (12)	O3—C12—C13	118.27 (16)
C5—N2—Zn2	107.49 (10)	C12—C13—H13A	109.5
C6—N3—H3A	117.2 (13)	C12—C13—H13B	109.5
C6—N3—H3B	122.8 (13)	H13A—C13—H13B	109.5
H3A—N3—H3B	119.2 (19)	C12—C13—H13C	109.5
N1—C1—C2	125.99 (16)	H13A—C13—H13C	109.5
N1—C1—Cl1	115.41 (13)	H13B—C13—H13C	109.5
C2—C1—Cl1	118.60 (14)	O6—C14—O5	125.42 (16)
C3—C2—C1	117.92 (16)	O6—C14—C15	117.28 (16)
C3—C2—H2	121.0	O5—C14—C15	117.30 (15)
C1—C2—H2	121.0	C14—C15—H15A	109.5
C2—C3—C4	118.36 (16)	C14—C15—H15B	109.5
C2—C3—C9	120.18 (16)	H15A—C15—H15B	109.5
C4—C3—C9	121.44 (16)	C14—C15—H15C	109.5
C5—C4—C3	118.00 (15)	H15A—C15—H15C	109.5
C5—C4—C8	115.72 (15)	H15B—C15—H15C	109.5
C3—C4—C8	126.28 (16)	C10—O1—Zn1	146.79 (11)
N1—C5—N2	112.32 (14)	C10—O2—Zn2	116.68 (11)
N1—C5—C4	123.46 (15)	C12—O3—Zn2	114.47 (11)
N2—C5—C4	124.21 (15)	C12—O3—Zn1	134.69 (11)
N3—C6—N2	119.47 (15)	Zn2—O3—Zn1	107.19 (5)
N3—C6—C7	120.21 (16)	C14—O5—Zn1	133.41 (11)
N2—C6—C7	120.32 (15)	C14—O6—Zn2^i^	128.40 (12)
			
O3—Zn2—N2—C6	−112.51 (15)	C11—C10—O1—Zn1	177.44 (14)
O2—Zn2—N2—C6	12.43 (16)	O3—Zn1—O1—C10	18.1 (2)
O6^i^—Zn2—N2—C6	118.98 (15)	O3^i^—Zn1—O1—C10	−161.9 (2)
O3—Zn2—N2—C5	55.10 (13)	O5^i^—Zn1—O1—C10	−73.1 (2)
O2—Zn2—N2—C5	−179.96 (11)	O5—Zn1—O1—C10	106.9 (2)
O6^i^—Zn2—N2—C5	−73.41 (12)	O1—C10—O2—Zn2	10.3 (2)
C5—N1—C1—C2	−0.7 (3)	C11—C10—O2—Zn2	−168.04 (12)
C5—N1—C1—Cl1	179.79 (13)	O3—Zn2—O2—C10	−39.80 (14)
N1—C1—C2—C3	−0.1 (3)	O6^i^—Zn2—O2—C10	69.02 (13)
Cl1—C1—C2—C3	179.40 (14)	N2—Zn2—O2—C10	−173.03 (12)
C1—C2—C3—C4	0.5 (3)	O4—C12—O3—Zn2	16.8 (2)
C1—C2—C3—C9	179.64 (16)	C13—C12—O3—Zn2	−163.00 (14)
C2—C3—C4—C5	−0.1 (2)	O4—C12—O3—Zn1	−138.26 (16)
C9—C3—C4—C5	−179.27 (16)	C13—C12—O3—Zn1	41.9 (3)
C2—C3—C4—C8	179.11 (17)	O2—Zn2—O3—C12	−106.56 (12)
C9—C3—C4—C8	0.0 (3)	O6^i^—Zn2—O3—C12	146.38 (12)
C1—N1—C5—N2	−178.05 (15)	N2—Zn2—O3—C12	13.12 (15)
C1—N1—C5—C4	1.1 (3)	O2—Zn2—O3—Zn1	55.16 (7)
C6—N2—C5—N1	178.72 (15)	O6^i^—Zn2—O3—Zn1	−51.90 (7)
Zn2—N2—C5—N1	9.14 (17)	N2—Zn2—O3—Zn1	174.84 (5)
C6—N2—C5—C4	−0.5 (2)	O5^i^—Zn1—O3—C12	−148.65 (17)
Zn2—N2—C5—C4	−170.03 (14)	O5—Zn1—O3—C12	31.35 (17)
C3—C4—C5—N1	−0.7 (3)	O1^i^—Zn1—O3—C12	−61.86 (17)
C8—C4—C5—N1	179.97 (16)	O1—Zn1—O3—C12	118.14 (17)
C3—C4—C5—N2	178.36 (15)	O5^i^—Zn1—O3—Zn2	55.03 (6)
C8—C4—C5—N2	−1.0 (3)	O5—Zn1—O3—Zn2	−124.97 (6)
C5—N2—C6—N3	−178.89 (16)	O1^i^—Zn1—O3—Zn2	141.82 (6)
Zn2—N2—C6—N3	−12.4 (2)	O1—Zn1—O3—Zn2	−38.18 (6)
C5—N2—C6—C7	1.3 (2)	O6—C14—O5—Zn1	−10.3 (3)
Zn2—N2—C6—C7	167.75 (12)	C15—C14—O5—Zn1	169.19 (11)
N3—C6—C7—C8	179.50 (17)	O3—Zn1—O5—C14	−137.70 (15)
N2—C6—C7—C8	−0.7 (3)	O3^i^—Zn1—O5—C14	42.30 (15)
C6—C7—C8—C4	−0.8 (3)	O1^i^—Zn1—O5—C14	−47.81 (15)
C5—C4—C8—C7	1.6 (3)	O1—Zn1—O5—C14	132.19 (15)
C3—C4—C8—C7	−177.70 (17)	O5—C14—O6—Zn2^i^	−5.4 (3)
O2—C10—O1—Zn1	−0.9 (3)	C15—C14—O6—Zn2^i^	175.15 (11)

Symmetry codes: (i) −*x*+1, −*y*+1, −*z*+1.

### Hydrogen-bond geometry (Å, °)

**Table:**

*D*—H···*A*	*D*—H	H···*A*	*D*···*A*	*D*—H···*A*
N3—H3B···O2	0.85 (2)	2.15 (2)	2.921 (2)	150.0 (17)
N3—H3A···O5^ii^	0.87 (2)	2.09 (2)	2.958 (2)	171.0 (19)

Symmetry codes: (ii) −*x*+2, −*y*+2, −*z*+1.
